# The Structural Dynamics, Complexity of Interactions, and Functions in Cancer of Multi-SAM Containing Proteins

**DOI:** 10.3390/cancers15113019

**Published:** 2023-06-01

**Authors:** Christopher M. Clements, Morkos A. Henen, Beat Vögeli, Yiqun G. Shellman

**Affiliations:** 1 Department of Dermatology, School of Medicine, University of Colorado Anschutz Medical Campus, Aurora, CO 80045, USA; christopher.m.clements@cuanschutz.edu; 2 Department of Biochemistry and Molecular Genetics, School of Medicine, University of Colorado Anschutz Medical Campus, Aurora, CO 80045, USA; morkos.henen@cuanschutz.edu (M.A.H.); beat.vogeli@cuanschutz.edu (B.V.); 3 Charles C. Gates Regenerative Medicine and Stem Cell Biology Institute, University of Colorado Anschutz Medical Campus, Aurora, CO 80045, USA

**Keywords:** disordered SAM domains, Eph receptors, LAR-RPTP, Liprin, CASKIN, SASH1, cancer, metastasis, tumor suppression

## Abstract

**Simple Summary:**

Cancer is a leading cause of death worldwide, with most of these deaths being the result of tumor metastasis (spread of cancer cells from the primary site). The sterile alpha motif (SAM) domain is a crucial protein module that can regulate many interactions among proteins, including those important for cancer development or metastasis. This review explores the literature on a group of under-studied proteins that contain multiple SAM domains. Our focus will be on these proteins, with a particular emphasis on the latest findings regarding the structural dynamics and interaction arrangements present within their SAM domains. We will also discuss the similarities as well as the uniqueness of their effects, functions, and regulations. We aim to provide a better understanding of these SAM domains and these proteins, which may offer clues to develop novel anticancer drugs.

**Abstract:**

SAM domains are crucial mediators of diverse interactions, including those important for tumorigenesis or metastasis of cancers, and thus SAM domains can be attractive targets for developing cancer therapies. This review aims to explore the literature, especially on the recent findings of the structural dynamics, regulation, and functions of SAM domains in proteins containing more than one SAM (multi-SAM containing proteins, MSCPs). The topics here include how intrinsic disorder of some SAMs and an additional SAM domain in MSCPs increase the complexity of their interactions and oligomerization arrangements. Many similarities exist among these MSCPs, including their effects on cancer cell adhesion, migration, and metastasis. In addition, they are all involved in some types of receptor-mediated signaling and neurology-related functions or diseases, although the specific receptors and functions vary. This review also provides a simple outline of methods for studying protein domains, which may help non-structural biologists to reach out and build new collaborations to study their favorite protein domains/regions. Overall, this review aims to provide representative examples of various scenarios that may provide clues to better understand the roles of SAM domains and MSCPs in cancer in general.

## 1. Introduction 

Cancer is a leading cause of death worldwide, accounting for nearly 10 million deaths in 2020, or nearly one in six deaths [1]. It has remained the second leading cause of death in the United States from 2011 to 2020 [2,3]. The sterile alpha motif (SAM) is a conserved domain found in a diverse range of proteins, including many signaling and scaffolding molecules involved in cancer development and progression. Multiple studies suggest SAM domains are an attractive target for developing cancer therapies. Thus, a better understanding of the structural dynamics, regulation, and functions of SAM domains may provide clues to develop anticancer drugs.

The SAM domain is a structurally conserved protein module, typically around 70 amino acids in length and comprises a five helical bundle [4,5,6,7]. SAMs are important in mediating protein–protein interactions (PPIs) [5,6,8,9], lipid interactions [10,11], and nucleic acid binding [12] (Table 1). In addition, SAMs are widely associated with scaffolding in signaling pathways [6,8,9,13,14,15]. Most of these proteins contain one SAM domain, but a select few have multiple SAM domains, which we refer to as multi-SAM containing proteins (MSCPs). These include the SASH1 and SARM1 proteins, as well as the protein families of ANKS1, Liprins, and CASKINs [4,8,14,16,17,18,19]. Many of these proteins have been reported to have roles in cancers and have been proposed as prognostic indicators in cancers. Although a few reviews on SAM domains have already provided comprehensive summaries on the structural features, the interactions, and roles in various diseases, these reviews have focused primarily on the single-SAM containing proteins [20,21]. Thus, the review here will focus on the SAM domains in the proteins containing multiple SAM domains. We aim to provide a comprehensive understanding of the structural dynamics, regulation, and molecular mechanisms of these domains, particularly in the context of cancer.

SAM domains are often expected to be well-folded and structurally stable in solution, interacting with partners via predictable binding interfaces [20,21]. Indeed, many SAM domains are known to multimerize, in both hetero- and homo-fashions, as dimers, oligomers, and even polymers. The study in solution of some of them, such as the tankyrase family, has been complicated by this polymerization [5,6]. Human genome-wide biochemical studies have also been carried out to better understand the SAM domain ‘polymerizome’ [4], yielding excellent information on several previously unstudied SAM domains.

However, SAM domain structural dynamics have been little discussed in the literature. For example, structural disorder has been observed for several individual SAM domains and may serve a regulatory function [7,17,18], yet few studies have explicitly investigated these dynamics. Interestingly, most of these appear to be in proteins with multiple SAM domains. This review will expand the discussion of the structural dynamics of SAM domains.

SAM domains are versatile and important in PPIs, protein–lipid interactions, and even nucleic acid binding [21]. They have been implicated in a variety of cellular processes, including signaling, transcriptional regulation, scaffolding, and cytoskeletal organization. With SAM domains serving such a vast array of functions, the study of SAM domains can be challenging [24]. In this review, we also provide a simple outline of methods and strategies for studying SAM domains, which can be applied broadly to the studies of single or multi-SAM domains, as well as other domains/regions. We aim to provide a framework for non-structural biologists to reach out to structural biologists and build new collaborations to study their favorite protein domains/regions, SAM or not.

Overall, we aim to give an overview of the structural dynamics, complexity of their interactions, and the functions in cancer of these MSCPs, an understudied group of proteins. The information here may provide clues to better understand the role of SAM domains in cancer in general.

## 2. Domain Architectures of the MSCPs 

**Figure 1:**
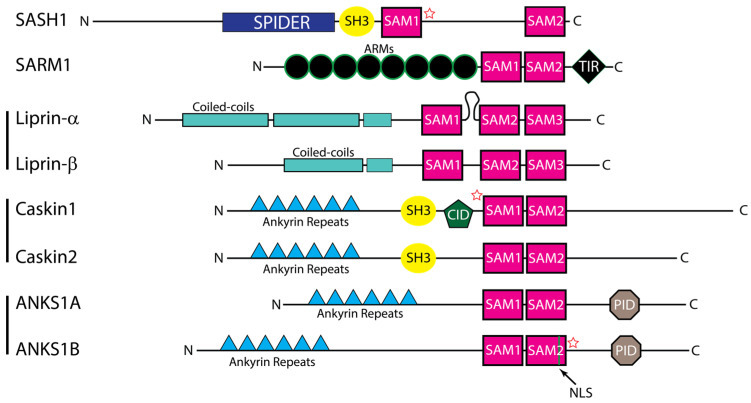
Domain architecture of MSCP proteins and families. Proteins are aligned by final SAM domain. Sizes are relative and not to scale. Representative protein members are shown here for each family. Stars indicate SAM domains with experimentally shown disordered states.

Proteins containing more than one SAM domain are relatively rare in the human proteome [4,14]. Based on their study [4] and the sequence similarity of these proteins, MSCPs can be grouped into five categories: SASH1 [25,26] and SARM1 proteins [16,27], and the protein families of Liprins [14,15,28,29,30], CASKINs [7,19], and ANKS1 [8,18] (Figure 1). Many of these MSCPs have important roles in tumorigenesis and/or tumor progression. Section 5 will discuss them in more detail, especially their major functions, their roles in cancer, their function and regulation, as well as how their SAMs regulate their functions.

All of these MSCPs are multidomain proteins with at least two SAM domains (Figure 1). Liprins are the only group that has three SAM domains. The most common structural arrangement of these multi-SAMs within the proteins is closely located to each other as tandems. The only exception is the two SAMs in the SASH1 protein, which are separated by nearly 500 amino acids. These differences in their arrangements likely affect how they function.

## 3. Structural Dynamics of the SAM Domains in MSCPs

To better illustrate the structural features of the SAM domains in MSCPs, we will start from the singular SAM domains in other proteins and compare the structural organization and dynamics between singular SAM and multi-SAMs in this section.

### 3.1. Structure and Interaction Arrangements of Singular SAM Domains

SAM domains are generally understood as wed and organized into a five-helix bundle with both *N*- and *C*-termini extending in the same direction away from the bundle [6,13]. The first four helices are shorter and bundled while helix 5 is the longest and passes across the four-helix primary bundle giving the characteristic structural appearance. SAM domains are most known for their ability to form oligomers of different sizes ranging from small dimers [18,22] to large, insoluble polymers [5,6]. These interactions can be those between the identical SAM of the same protein (homo-oligomer) or those between SAM domains of different proteins (hetero-oligomer).

These SAM–SAM interactions can have several arrangements: head-to-head, tail-to-tail, and most prominently head-to-tail [18,20,22]. These vary subtly from molecule to molecule but generally, the *N*-terminal head, consisting of the helices and respective loops of helix 2, 3, and 4, interacts with the *C*-terminal tail, consisting of helix 5 and occasionally helix 1. Thus, the faces of the head-to-tail arrangement are termed mid-loop (ML) and end-helix (EH).

### 3.2. Disordered and Unique Scaffolding in Multi-SAM Domains

#### 3.2.1. Intrinsic Disorder in Multi-SAMs and Protein Function

Unlike classical globular, structured proteins, intrinsically disordered proteins (IDPs) lack a well-organized hydrophobic core, often increasing the complexity of their PPIs and attaining their functionality in a different manner [31]. The elevated flexibility of IDPs or intrinsically disordered regions (IDRs) confers the ability to participate in multiple functions. This promiscuity is further enhanced by frequent post-translational modifications that increase the number of functional states for the protein [32,33]. Due to IDPs’ conformational variability and adaptability, they are frequently involved in the scaffolding and recruitment of different binding partners [34].

When IDPs are scaffolded by their partner proteins, the extended binding interface preserves the open structure of disordered assemblers, allowing a single IDP to bind to multiple proteins. Furthermore, disordered regions provide the advantage of reduced steric clashes upon binding that prevent the formation of comparably large complexes in the case of folded proteins. Once in complex, many IDPs adopt a defined fold and may form large complexes or even fibrils. Other IDPs maintain structural ambiguity even in complexes, fittingly referred to as ‘fuzzy complex’ [35].

**Figure 2:**
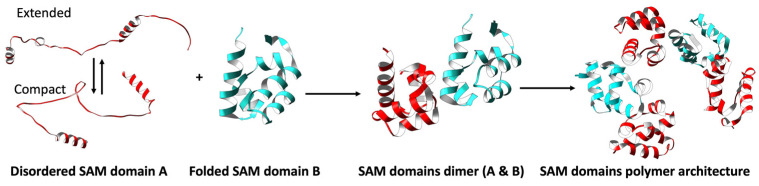
Binding of the disordered SAM domain in tandem with a well-folded SAM domain leads to a heightened level of complexity in the system. Schematic representation illustrating the potential impact of having two SAM domains in tandem. In isolation, the disordered SAM domain (domain A: depicted in red) exhibits higher conformational flexibility. However, upon interacting with a folded SAM domain (domain B: illustrated in blue), a dimer is formed, accompanied by folding of the disordered domain. This event may result in the formation of higher-order polymeric structures crucial for the functioning of the system.

Many SAMs in MSCPs act similarly as singular SAMs in that they can have wed structures and form multimers. However, many of these multi-SAM domains of the same protein display an interesting characteristic: when they are expressed individually in bacteria, one SAM is disordered and the other(s) well-folded [7,14,17,18]. Thus, many MSCPs appear to have one SAM domain with a disordered propensity in certain situations (see below and Section 5 of each MSCP for details). Further, having additional SAM domains in a single protein, and particularly in tandem, provides opportunities for additional intra-molecular interactions, unique restrictions, and increased complexity for protein behavior and function. As the system’s complexity increases, the disordered SAM domain can potentially fold upon interaction with the other SAM domain and result in a higher oligomeric structure (Figure 2).

We recently reported the first study that characterized a disordered SAM domain at the atomic level [17]. We used multiple biochemical and biophysical assays, including nuclear magnetic resonance (NMR), to characterize the SAM1 domain of SASH1. We found that the SAM1 domain in solution is mostly a disordered monomer with some helical propensity, with the presence of a minor population of an oligomeric state.

**Figure 3:**
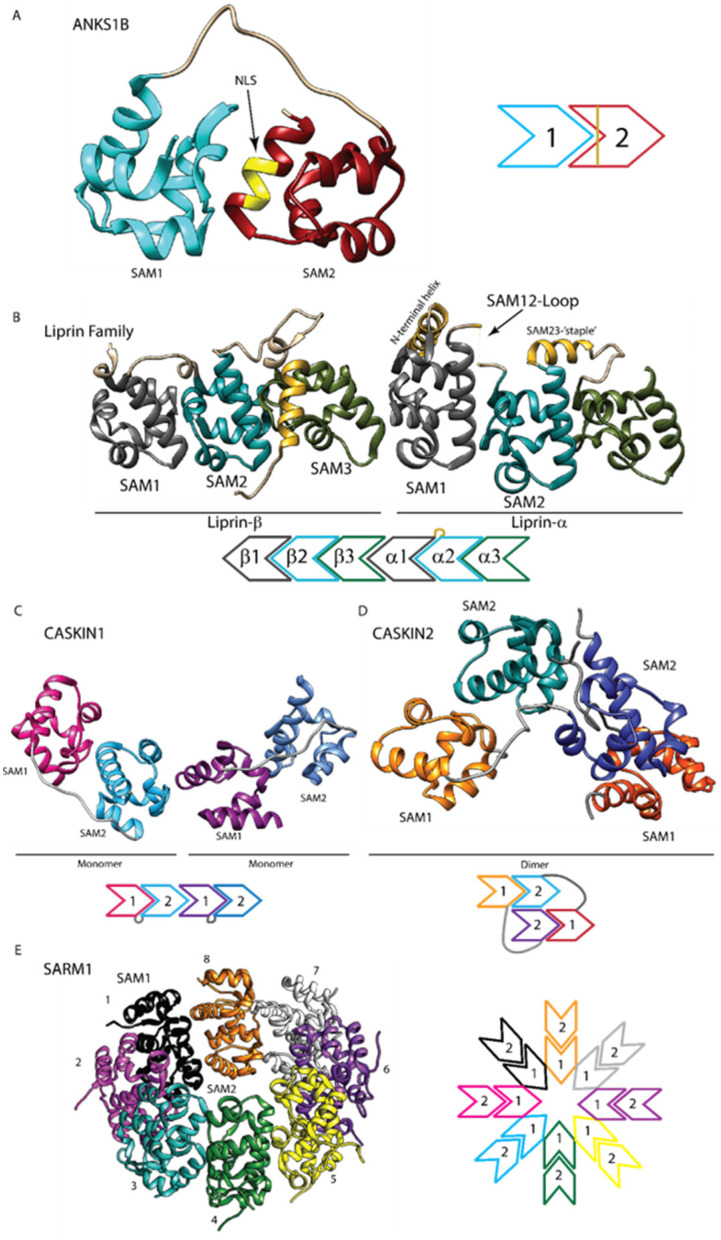
Diversity in multimerizations of the MSCPs’ SAMs. (**A**) The ANKS1B tandem SAM1/2 domain (PDB: 2KIV) forms an intramolecular head-to-tail monomer. SAM2 is disordered when not bound to SAM1, which may serve to regulate the nuclear localization sequence (yellow) in helix 5 of SAM2. (**B**) Liprin-α and Liprin-β each has three tandem SAM domains that intramolecularly trimerize (PDB: 3TAD). SAM3 of Liprin-β can dimerize with SAM1 of Liprin-α, creating a Liprin heterodimer and a linear SAM hexamer in a head-to-tail fashion. (**C**) CASKIN1 tandem SAM1/2 (PDB: 3SEN) forms intramolecular head-to-tail interactions that then extend to other CASKIN2 tandem SAM1/2 domains to form a helical oligomer of monomers. (**D**) The tandem SAM1/2 domain of CASKIN2 (PDB: 5L1M) forms intermolecular interactions to form a dimer that may expand to an oligomer of dimers. This “domain-swapped dimer” can lead to a branched oligomer in contrast to CASKIN1′s spiral oligomer. (**E**) The SARM1 tandem SAM1/2 (PDB: 6QWV) form intramolecular head-to-tail interactions that then utilize lateral interactions to form a stacked closed octameric ring.

Another example of a SAM that displays a disordered state in certain contexts is the SAM2 in ANKS1B [18]. ANKS1B has two tandem SAM domains, with a nuclear localization sequence (NLS) in helix 5 of the second SAM domain (Figure 3A). Both SAM1 alone and the tandem SAM domains were well-folded when expressed in bacteria, but SAM2 displayed poor structural features [18]. It has been proposed that SAM2 is stabilized by SAM1, and when this interaction is perturbed, such as with phosphorylation, SAM2 unfolds and exposes the NLS for protein nuclear transport [18].

Additionally, NMR analysis has shown that the SAM1 of CASKIN2 is disordered, while SAM2 and the tandem SAM domains are well-folded [7]. Similarly, the SAM2 of Liprin-α is supported by both SAM3 and a helical linker that ‘staple’ the two together (Figure 3B) [14]. Moreover, SAM1 of Liprin-α may be similarly stabilized by extensive hydrophobic contacts in α1 with an *N*-terminal α-helix (Figure 3B) [14]. A high-throughput study of SAM domains showed that many of the individual SAMs of MSCPs either are poorly expressed in their system or non-/weak-polymers [4], and it is possible that some may be due to their disordered features. This possibility of the disordered feature has not yet been well explored in SAM domains, even though this is quite important for regulating PPIs as stated above. Therefore, it is imperative to consider the potential presence of disordered features when investigating proteins that contain single or multiple SAM domains [7,14,17,18].

#### 3.2.2. Multi-SAM Domains Increase the Complexity of SAM-Mediated Oligomers

The arrangement of SAM-SAM interaction in multi-SAMs is similar to the one between singular SAMs, with the most common one of the head-to-tail. However, multi-SAMs provide the opportunity to have interactions not only intermolecularly (SAMs on different molecules) but also intramolecularly (SAMs within the same molecule). This can increase the complexity of the final complexes (Figure 3).

The oligomerizations of the CASKIN protein family, CASKIN1 and CASKIN2, are instructive examples. SAM1 and SAM2 in CASKIN1 form intramolecular head-to-tail complex of, and this protein monomer (SAM-dimers) then further oligomerizes intermolecularly with other CASKIN1 proteins to form an oligomer of protein monomers (Figure 3C) [19]. On the other hand, CASKIN2 forms a dimer with SAM1 of one molecule binding to the SAM2 of another molecule along two SAM1-SAM2 interactions, forming a “domain-swapped dimer” (Figure 3D) [7]. This dimer then further oligomerizes with other CASKIN2 proteins to form an oligomer of dimers. Interestingly, the CASKIN1 oligomer can only grow as a fibril in both directions, while the CASKIN2 oligomer can form a branched structure. Thus, having both inter- and intra-molecular interactions increases the complexity of the final oligomers [7,19].

Another example is SARM1, which forms intramolecular dimers of SAM1 and SAM2 which then seed the formation of a closed octamer ring (Figure 3D) [16]. This ring is additionally seen in the full protein with the inner ring being the SAM domains and the outer ring being the ARM and TIR domains [16,27]. Interestingly, the SAM1 domains appear to be more important in the formation of this octamer [16]. Of note, no individual expression of the SARM1-SAM domains was attempted, however, there is a short helix *N*-terminal of the first SAM domain forming a hydrophobic surface with alpha-3 and 5 of SAM1 [16]. This may be like the longer helix just *N*-terminal of the Liprin-α’s SAM1 domain, which is necessary for stabilizing the SAM1 fold [14].

The Liprins are yet another example of this complexity. In both the Liprins-α’s and Liprin-β’s, the three-tandem SAM domains (also known as the Liprin homology domain or LHD) [28] form linear trimers within the protein in a 1,2,3 progression (Figure 2B) [14]. This can then heterodimerize with Liprin-β via the SAM3^Liprin-β^ and the SAM1^Liprin-α^ (Figure 2A) [14]. This complex serves to bind with CASK and, in the absence of Liprin-β, LAR-RPTPs [14,30,36]. The Liprin SAMs thus form a versatile and unique protein–protein interaction that has been reviewed in greater depth in [36].

Taken together, the presence of a single SAM domain in a protein can suggest a multitude of functions with diverse molecular mechanisms within each. The incorporation of an additional SAM domain, possibly possessing disordered features, has the potential to exponentially increase the complexity of oligomerization and complex formation for that protein. Moreover, these SAMs may interact with diverse partners to increase the complexity even further. Thus, these SAMs can operate synergistically in the above MSCPs with many functions likely not even yet discovered.

## 4. Methods to Study the SAM Domains from MSCPs

**Figure 4:**
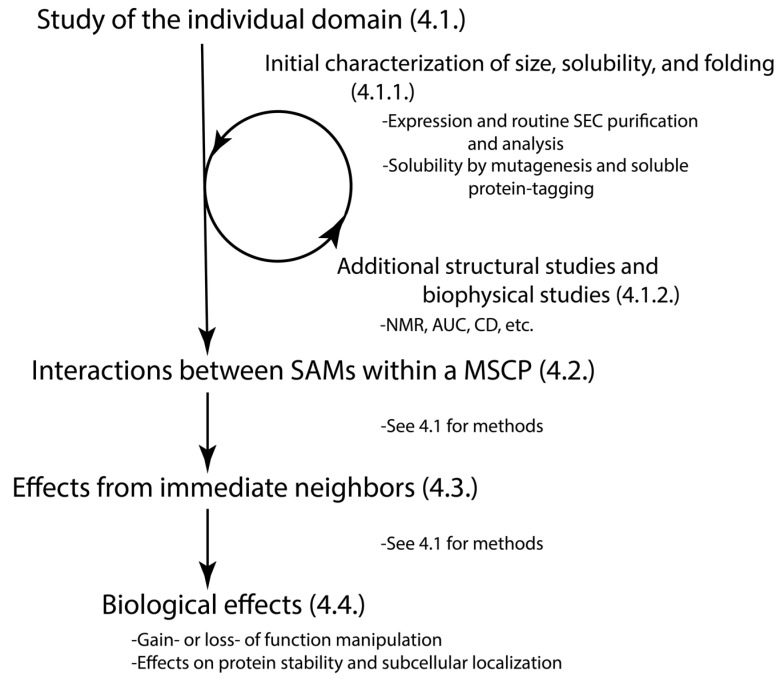
Schematic for approaching the study of MSCP SAM domains. The numbers at the end indicate the subsections.

To better understand the structural dynamics and the role of unstudied SAM domains, we suggest several methods and provide a flowchart (Figure 4). The objective of our work is to establish a framework for non-structural biologists to approach their research projects in a manner that enables them to reach out to the structural biologists and foster a collaborative effort to advance their research. The suggested flowchart and methods are based on the following questions that biologists would like to ask. Please note the methods described in Section 4.1 can be used in the rest of structural studies (Section 4.2 and Section 4.3). Keep in mind that the approach to their study should be tailored to the unique size, shape, and character of the domain.

### 4.1. Does the Individual SAM Domain Exist as a Well-Folded or Disordered Monomer, a Dimer, or an Oligomer in Solution?

#### 4.1.1. Initial Assessment on the Size, Solubility, and the Status of Folded vs. Disorder of the Individual SAM Domain

Many scientists have used pMAL and pET28a vectors to express the soluble SAM domains in bacteria and followed up with the routine purification by affinity and size-exclusion chromatography (SEC) [5,6,7,17,18,22]. Generally, a fusion tag such as a His_6_-tag can enable a rapid affinity-SEC purification. The final SEC of purified protein has been used as an initial assessment of size and the status of folded vs. disordered [14,17,36].

SEC provides information about the hydrodynamic radius of the sample such as whether the protein is a monomer or larger [37,38]. However, it can be difficult to distinguish between a disordered monomer and smaller oligomer, such as a dimer or trimer, as the disorder will make the monomer run larger [17]. Thus, these results should be followed up with a more rigorous methodology described below in Section 4.1.2.

However, not all domains will be soluble in this system. Insolubility of SAM domains is often due to large polymerization events [5,6]. A simple methodology would be to try expanding the domain boundaries. This worked in both the study of Liprin-α2 [14] and the study of SARM1 [16]. Alternatively, another way to overcome this is the mutagenesis of the key residues involved in polymerization, as performed in the studies of the SAM domain of tankyrases [5,6]. DaRosa et al. used computational modeling to predict key residues for substitution, while Mariotti et al. used sequence alignment to predict mutants to reduce polymerization [5,6]. Crystallography was then used to refine the mutants for solution experiments [6]. Indeed, SAMs of the CASKIN family oligomerize to a lesser extent than those in tankyrases, allowing mutant selection for improved solubility and crystallography to be used for structural determination [7,19]. Mutagenesis studies can further confirm the polymerization of the domain by removing important hydrophobic or electrostatic contacts [5,6,14,18,36]).

Another method to overcome the insolubility issue is to use a soluble protein tag to pull the polymer into solution for purification, such as the super charged negGFP (modified to have a net charge of −30) [4,39]. The GFP increases the visualization of the protein in crude extract while the charge improves solubility and ensures the reliable migration on native gels [4].

SAM domains in MSCPs, individually expressed, tend not to experience extensive polymerization [4]. Additionally, many seem to have at least one domain with disordered characteristics and one with more folded conformations [7,17,18]. Further studies are required to determine the role of this disordered propensity and what role it may play in the protein’s biological context.

#### 4.1.2. Additional Structural Studies of Individual SAM Domain

Each method has its advantages and limitations, and thus utilizing multiple methodologies will yield the most useful results. The commonly used methods include NMR [7,17,22,40], multi-angle light scattering (MALS) [17,37,38], analytical ultracentrifugation [22,37,41], X-ray crystallography [6,7,14,19,22,42] and electron microscopy [6,19].

NMR has been shown to be a highly versatile tool for studying and characterizing both well-folded [5,7] and disordered SAM domains [7,17]. While the high flexibility of disordered proteins presents a challenge for their study on a structural level, NMR has emerged as a powerful technique for characterizing these proteins at an atomic level by providing valuable information about the chemical environment and dynamics of these proteins and their interactions with other proteins [43].

The relatively small size (~70 amino acids) of SAM domains makes them ideal to be studied by NMR as a monomer or a small oligomer. In the case of folded SAM domains, NMR has provided valuable information about the multiple conformation structure of these domains and their interactions with their binding partners [8,9,18,22]. In addition, NMR has been successfully used to characterize many heterotypic SAM–SAM complexes in solution [7,8,9,18].

NMR will provide basic structural information from both 1D and 2D experiments. Peak dispersion will provide information on whether the protein is folded or disordered while peak intensities will offer information on population and dynamic behavior [17]. For example, absence of dispersed peak may be indicative of the major population being disordered [17]. Additionally, larger proteins suffer from disfavorable higher relaxation rates in NMR which result in signal broadening or disappearance [44]. Thus, the dispersed peaks in the 2D NMR spectrum of SAM1 of SASH1 was due to more ordered segments in the otherwise disordered monomer rather than a larger folded oligomer [17].

For determining the sizes of the purified products, SEC-MALS can provide information on the molecular weight of different populations [17]. In addition, analytical ultracentrifugation can provide information on the stoichiometry, compactness, mass, and hydrodynamic radius [22].

X-ray crystallography and electron microscopy are also useful methods in certain situations [4,7,19]. Crystallography is an excellent tool for looking at homologous structures that are able to form crystals, which can yield high resolution structures of proteins and their complexes. The challenges come predominantly from the crystallization process. Indeed, the requirement for homologous samples eliminates the possibility of using it for disordered proteins as they will not crystallize. For a more macrological level, electron microscopy is an excellent method for observing large oligomers and polymers as seen in the CASKIN1 and the mono-SAM containing tankyrase 2 [6,19].

For many of these techniques, experiments should be conducted under additional conditions, including different protein concentrations, buffer salt concentrations, and temperatures. The varying protein concentrations will provide information on homo-multimerization potentials [22]. Buffer salt concentrations will offer details on the role of electrostatic interactions in any self-multimerization events [19]. Further, the temperature can be changed, especially in NMR, to change the solution dynamics of the sample and to improve signal resolution [7,17]. Moreover, circular dichroism (CD) is a rapid, simple, and useful method for calculating the overall secondary structural content of proteins [45]. CD can be used for heat, salt, and concentration profiling to determine any changes to the secondary structure.

These structural details offer valuable insight into the molecular functions of these SAM domains. A thorough analysis of the binding mechanism of SHIP2 and ANKS1A with EphA2 identified the rules governing the interactions and discovered SAMD5 as a novel binding partner of Eph receptors [42].

### 4.2. How Do the Multiple SAM Domains within the Protein Interact with Each Other?

In proteins with multiple SAM domains, their ability to interact should be explored. This can be undertaken using the above methods and by titrating one SAM into the other. Ideally, the methods in Section 4.1 would be used for both domains prior to this step. This is important primarily for two reasons. If SAM domains are separated by very large linkers, such as in SASH1, it would be impractical to study them in full length context with NMR, due to the size constraints. Additionally, tandem SAM domains are often linked by short linkers and the removal of these linkers by separate expression will provide information about the importance of the linker in multimerization [14]. Molecular dynamics has emerged as an effective tool for studying heterotypic SAM–SAM interactions, like those in the EphA6-ANKS1A complex [46]. The authors employed this approach to map the binding process and identify crucial binding residues. Furthermore, their calculated standard binding free energy closely aligned with the previously published experimental value [42].

### 4.3. How Do the SAM Domains Exist in the Context of Their Immediate Neighbors and Full Proteins?

In many tandem SAM domains, co-expression has had stabilizing effects on SAMs with disordered propensity [7,18]. In these cases, the more well-folded SAM ‘stabilizes’ the folded state of the more disordered SAM domain. However, this is not solely reserved for tandem SAM domains. It has been shown that adjacent and linker helical motifs are responsible for stabilizing the structural fold in the Liprin family [14]. Thus, it is important to expand the sequence beyond the SAM domain in certain cases to determine if additional structural motifs may be stabilizing the helical fold if all other methods fail to induce structural characteristics. Additionally, an adjacent non-SAM domain may encourage domain folding. If this is the case, an overlay of 2D-HSQC spectra of isolated domains with the 2D-HSQC of the tandem domains will highlight regions that are perturbed by the presence of the linker and adjacent domain(s) by chemical shift changes [7,47].

The influence of a domain may also expand beyond the immediate neighbors, and structural dynamics or mutagenesis may induce long distance changes. Scaffold proteins are often large and heterologous in solution, making them challenging to study globally by traditional structural methods such as NMR, X-ray crystallography, and electron microscopy. Because of these challenges, the use of small-angle X-ray scattering (SAXS) and/or small-angle neutron scatter (SANS) can be useful for observing these large ensembles at low resolution in solution. The combination of this with the use of higher resolution techniques on the well-folded domains can offer insights into both the local and global effects of mutations or conformational changes. A recent study of the NHERF1 scaffold protein combined NMR for domain level analysis with SANS to extrapolate the effects of single amino acid substitutions on global dynamics [48]. Three disease variants from individual amino acid substitutions were found to alter the global conformation of the protein and significantly alter the local PDZ domain structure [48]. Global structural studies such as this would lend valuable information on the long-range allosteric changes from protein dynamics and clinical variants.

### 4.4. How Do the SAM Domains Regulate the Protein Function? 

This part is very context dependent, with specific assays relevant to the protein activity and biological functions. However, there are several useful general approaches. First, gain- or loss-of-function studies with genetic manipulations for the specific SAM domain(s) will provide mechanistic insight. One excellent example is the structural-function investigation of SAM domains in SARM1’s functions [49]. To examine which domains of SARM1 are critical for its role in promoting axon degeneration, Gerdts et al. constructed various mutants of SARM1, which contain deletions or missense mutations of SAMs or other domains [49]. They then determined the effects of these mutated SARM1s on its ability to multimerize with biochemical structural studies, and on the SARM1’s ability to promote axon degeneration with biological assays.

Secondly, SAM domain-mediated interaction can also regulate protein localization and stability, and thus immunoblot and immunostaining can be used. For example, fluorescence microscopy was used to determine the effects of SAM variants in the CASKINs on the proteins’ subcellular localization [7,19]. These MSCPs are involved in the reorganization of the cytoskeleton, and both CASKINs were shown to polymerize in solution. The polymerization was maintained in the wild type with puncta formation in the cells and loss of puncta protein distribution in non-polymerizing variants [7,19].

In sum, combinations of biochemical, biophysical analyses, genetic manipulations, and the relevant biological assays will provide a comprehensive understanding of the structural dynamics, regulation, and biological functions of any domains or regions.

## 5. MSCPs’ Major Functions, Their Roles in Cancer, and the Structural Regulation of Their SAMs

### 5.1. Overview

MSCPs can be grouped into proteins SASH1 and SARM1, plus the protein families of ANKS1, Liprins, and CASKINs. All are multidomain proteins with scaffolding capacity of diverse binding partners. Thus, they have many functions and regulations, and it is beyond the scope of this review to summarize all. Therefore, this section will focus on MSCPs’ major functions, their roles in cancer, and how their SAM domains affect their main biological functions. The goal here is to provide representative examples of various scenarios that may provide clues to better understand the roles of SAM domains and these proteins in cancer in general.

Many similarities exist among the members of MSCPs. In terms of the effects on cancers, cell adhesion, migration, and metastasis are the main ones. Specifically, both SASH1 and Liprins can regulate these functions in various cancer cell types, likely through modulating proteins involved in cytoskeleton organization [25,50,51,52,53,54,55]. In addition, the ANKS1 family can regulate cell migration and adhesion indirectly, through regulating Eph receptors (erythropoietin-producing hepatocellular receptors) signaling [56,57,58,59,60]. In terms of signaling pathways, all are involved in some types of receptor-mediated signaling. Specifically, both SASH1 and SARM1 are involved in the TLR pathways of innate immune response [61,62,63]; ANKS1A/B are modulators and downstream targets of Eph receptors [8,18]; and Liprins and CASKINs are downstream of LAR-RPTPs (Leukocyte common antigen-related receptor tyrosine phosphatases), best known for their ability to regulate neuronal development and synaptic adhesion pathways [15,19,30,55]. The Liprins interact directly while CASKIN1 operates further downstream via its binding with CASK [15,19,29,30,55,64]. Further, many of these proteins seem to be involved in neurology- or brain-related functions or diseases. For example, both the Liprins and CASKINs families play crucial roles in synapse organization and function [7,15,23], and SARM1 is one of the main mediators for axon degeneration [65]. ANKS1B is mainly expressed in brain, and genome-wide associated studies identified *ANKS1B* gene as a top locus associated with drug response in central nerve system [66]. SASH1 is expressed in brain tissues, and its expression in glioma tissues is positively correlated with better postoperative survival in patients [67]. However, each group of MSCPs has their own functions and regulations.

### 5.2. SASH1 

SASH1 (SAM and SH3 domain-containing protein 1) is a tumor suppressor, belonging to the family of the SASH1/SLy adaptor/scaffold proteins [51]. However, SASH1 is the only one in this family with two SAM domains; the other two members (SAMSN1 and SASH3) only contain one SAM domain. All three proteins have a conserved region containing one SH3, one SAM, and a newly defined region, SLy Proteins Associated Disordered Region (SPIDER) [26]. SASH1 is a scaffold protein with context-dependent biological functions, including suppression of tumor metastasis [50,52,68,69], lung development [70], and pigmentation [71,72,73,74,75,76,77,78,79].

In cancer cells, SASH1 can inhibit proliferation, migration, and metastasis. Deletion of the SASH1 gene in tumor samples significantly correlates with poor patient survival in the TCGA pan-cancer data set [51]. Studies with colon and breast cancer patients also indicated down-regulation of SASH1 associates with metastasis [25,51,80]. In vitro studies with cancer cells show that SASH1 protein inhibits migration, invasion, and epithelial-mesenchymal transition (EMT) [50,69,81,82,83], decreases cell proliferation and survival [50,84,85], or promotes apoptosis [86] in breast, colon, melanoma, and many other cancer types. In a mouse xenograft model, knocking down SASH1 in colon cancer cells blocks metastasis [69]. SASH1 plays a role in actin dynamics and regulates cytoskeletal reorganization and integrin-mediated cell adhesion [51,52,69], which likely contributes to its role in suppressing tumor metastasis. In term of signaling, SASH1 has the ability to regulate several signaling pathways downstream (FAK [50], NOTCH1 [87], PI3K, SHH-GLI1 [82], TGFB1 [88], CRKL/SRC [69]. Taken together, SASH1 in tumor cells can be a tumor suppressor for multiple cancers.

SAM domains in SASH1 are crucial for SASH1’s functions and have been implicated in multiple biological functions and diseases. SAM1 is found to be part of the region important for SASH1’s function in regulating cytoskeleton reorganization in cancer cells [52]. Pathologically, a p.R644W variant in Helix 1 of SAM1 is associated with a human dermatological disorder [78]. Further, the deletion of SAM1 disrupts the interaction of SASH1 with β-arrestin 1, which is important for lung development through regulating the AKT and the endothelial nitro oxide signaling pathways [70].

Structurally, SASH1 was included in RIKEN’s high-throughput structural proteomic pipeline, yielding NMR solution structures of the SH3 (PDB: 2EBP) and SAM2 domains (PDB: 2DL0). Although there is no supporting methodologies of protein expression and purification, the solution structure of SAM2 suggests that this domain is (1) well-folded and highly structured in solution and (2) can be expressed and purified in reasonably high yields. High throughput analysis identified both SAM1 and SAM2 of SASH1 as non-/weak polymers [4]. This supports the RIKEN solution structure of SAM2 as being monomeric. Neither a solution nor crystal structure of SAM1 is published in the PDB, suggestive of more complicated protein dynamics at play. Indeed, SAM1 is found to be disordered in solution with a small portion oligomerizing into tetramers and most forming a dynamic ensemble of predominantly disordered structures [17]. Further analyses of the p.R644W substitution showed no shift in size in SEC or in the HSQC fingerprint [17]. However, a dual D663A/T664K substitution in helix 3 resulted in a significant shift in the oligomeric state population, increasing it from 1% to approximately 50% [17]. One explanation for this is that SAM1 samples transient structured states (the classical helical bundles of SAM domains, for example) and this substitution removes the acidic residue at 663 and introduces a basic residue at 664 that is hypothesized to produce a salt bridge with E682 on the *N*-terminal of helix 5. This suggests that the acidic D663 and the neutral T664 are essential in preventing an otherwise preferred SAM1 homo-oligomerization.

So far, experimental evidence demonstrates that neither SAM1 nor SAM2 of SASH1 substantially homo-polymerize. Interestingly, SASH1 is the only protein with multiple SAM domains separated by over 400 residues, instead of tandem SAM domains clustered together in the other cases listed in this review. However, the two SAM domains in SASH1 do display some similar phenotypes with those tandem SAM domains: when individually in solution, one (SAM1) is disordered while the other (SAM2) is well-folded.

As of this writing, no study has determined whether SAM1 and SAM2 interact. When tandem SAM domains in other MSCPs are expressed together, they produce compact and well-folded domains. In SASH1, perhaps nearby SH3 stabilizes the fold of SAM1. Indeed, SAM1 is in the conserved tri-domain region of SPIDER-SH3-SAM1, important for SASH1′s function and conserved across the SLy protein family [52]. Perhaps the two SAMs interact in an inter- or intramolecular fashion as a form of self-regulation. The disordered structure of SAM1 may play a role in SASH1’s interaction with β-arrestin whereas multimerization with SAM2 could regulate this interaction. Further studies will be necessary to tease out the molecular mechanism.

**Figure 5:**
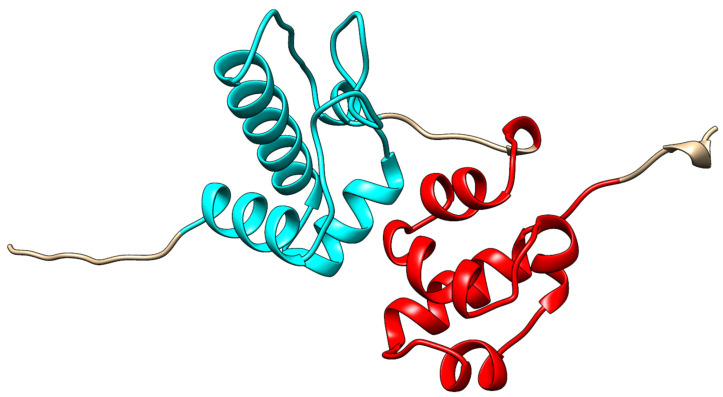
The Alpha-Fold predicted structure of SASH1′s *C*-terminal (1082–1247). This includes the SAM2 domain (1177–1241; red) and a novel SAM-like helical bundle (1092–1170; cyan).

However, perhaps there is a missing piece to the puzzle. Interestingly, Alpha-Fold [89] predicted the structure of SASH1 to include a helical bundle immediately *N*-terminal to the SAM2 (1177–1241) domain, with the two appearing to be in a bound tandem structure, not unlike that of the tandem SAM domains discussed in this review (Uniprot: O94885, structure: AF-O94885-F1). This potential SAM-like domain (1092–1170; Figure 5) has never been reported and could just as easily be a computational artifact as a novel domain. As of this writing, SASH1 is the only human protein with multiple SAM domains that are not in tandem, suggestive of either a unique function and mechanism of SASH1 or that key structural information for the function of SASH1 may be missing. If this SAM-like domain is confirmed to exist, it would indicate that SASH1 does indeed have tandem SAM-like domains that would put it in a similar class as the other proteins discussed in this review. Interestingly, the SAM-like domain is located near the reported proline rich region (984–989) known to bind the SH3 of CRKL [69].

### 5.3. ANKS1 Family

The Ankyrin repeat and SAM domain-containing protein 1 family (ANKS1) is a subgroup of phosphotyrosine-binding proteins, including ANKS1A and ANKS1B. Many names have been used for them, making the literature search complicated, including Odin and ANS1-A for ANKS1A, and AIDA-1, EB-1, ANKS2, and Cajalin-2 for ANKS1B. For clarity, we are referring to them as ANKS1A and ANKS1B in this review. Both contain two tandem SAM domains, a phosphotyrosine-interaction domain (PID), and multiple ankyrin (ANK) repeats [8,18]. The presence of the phosphotyrosine-interaction domain (PID) indicates their involvement in kinase-mediated signaling pathways. Indeed, this family has been reported as both regulators and downstream targets of several cancer-associated receptor tyrosine kinases, including Eph receptors (erythropoietin-producing hepatocellular receptors) [56,57,58,59,60]. Eph receptors can regulate tumor cell proliferation, migration, angiogenesis, and metastasis; however, their effects can be pro- or anti-tumorigenesis, depending on the context [90]. ANKS1A and ANKS1B have been reported to impact tumorigenesis through their interaction with these receptor tyrosine kinases.

ANKS1A normally expresses ubiquitously in many tissues and cell types. The reported roles of ANKS1A in cancers are mediated through several receptor tyrosine kinases. In addition to Eph receptor mentioned above, it is also a downstream mediator of EGFR or SRC family kinase signaling in cancer cells [56,57], two well-known activated signaling pathways in cancers critical for tumorigenesis. On the other hand, ANKS1A also can regulate these pathways. For example, ANKS1A regulates the stability and recycling of EGF receptor [58], the degradation of EphA8 and EphA2 in mouse embryonic fibroblast [59], and COPII-mediated anterograde transport of EphA2 and ErbB2 in a colon carcinoma cell line [60].

ANKS1B is mainly expressed in the brain [91], which may limit its potential roles in a broad range. Its best-known function is a scaffold protein modulating synaptic transmission and plasticity, involved in developmental disorders to Alzheimer’s disease [92]. Very few studies investigated its role in cancer. Dysregulation of its expression was found in smoking-related clear cell renal cell carcinoma [93], and a few single nucleotide polymorphisms in ANKS1B have been associated with cancers [84]. Intriguingly, several studies identified the pro-cancer roles of the circular RNA of ANKS1B (cANKS1B), which is a circular RNA originating from exon 5–8 of the ANK1B gene. It is proposed to act as a sponge for various cell-type specific miRNAs and thus influence various important pro-cancer gene expressions [94,95,96,97]. For example, in triple-negative breast cancer, cANKS1B is significantly upregulated, and functional assays revealed that it induces EMT, and promotes breast cancer in vitro and in vivo, and this is through sponging miR-148a-3p and miR-152-3p to increase USF1 and TGF-β1 [94]. Similar effects have been reported in other cancers with different mediators, and these include colorectal cancer [95], prostate cancer [96], and oral squamous cell carcinoma [97].

SAM domains in the ANKS1 family play important roles in their functions. One example is the role of ANKS1A’s (Odin) SAM domains in the stability of EphA8 and EphA2 [59]. These SAM domains bind ubiquitinated EphA8, and overexpressing ANKS1A protects EphA8 and EphA2 from degradation following ligand stimulation and promotes EphA-mediated inhibition of cell migration. Importantly, a SAM domain deletion mutant of ANKS1A causes dominant-negative effects on these functions of endogenous ANKS1A. Although biochemical and structural studies suggested that its SAMs play important roles in the binding to EphA2 [8], loss-of-function studies in CT26, a colon carcinoma cell line, did not find a significant role of these SAMs in the transport of EphA2. Specifically, deletion of the PID domain but not its tandem SAM domains alters the intracellular ANKS1A/EphA2 complex [60]. These results indicate the importance of loss-of-function studies with cells. Further studies are needed to investigate how SAM domains in this family affect their roles in cancer.

Structurally, the SAM domains of ANKS1B are better investigated. These tandem SAMs can bind intramolecularly in the typical head-to-tail arrangement, with the ML of SAM1 binding to the EH face of SAM2 [18]. When expressed individually in *E. coli*, SAM1 appears as a stable monomer while SAM2 as “aggregate and be only partially folded” [4,18]. These results support the concept that a well-folded SAM1 domain stabilizes a disordered SAM2 in solution.

One interesting feature of ANKS1B’s SAMs is that SAM2 contains a nuclear localization signaling (NLS) region. Solution NMR studies of the tandem SAM domains showed that they bind intramolecularly. It was proposed that post-translational modifications may separate the tandem SAM domains and induce the unfolding of SAM2, resulting in the exposure of the NLS [18]. If true, this would be an intriguing example of structurally mediated regulation in tandem SAM domains. Further investigations are needed to unravel the regulatory mechanisms.

### 5.4. Liprins

The liprins are multidomain proteins, generally consisting of *N*-terminal coiled-coil regions interspersed with disordered regions and *C*-terminal tandem SAM domains (Figure 1). These MSCPs have three SAM domains, which are located in a conserved region of liprins, also known as the Liprin Homology Domain (LHD). They include four liprin-α proteins (α1, α2, α3 and α4), two liprin-β proteins (β1 and β2), and Kazrin E. Liprins, which are a family of scaffold proteins known to function in several important neurological functions related to cell signaling and organization of synaptic structures [98]. Kazrin E. is best known for its role in desmosome assembly and tissue morphology [99].

The liprins are members of the proteins that interact with LAR-RPTPs, which are key synapse organizers in neuronal development [98]. LAR-RPTPs mediate various synaptic adhesion pathways through interactions with a host of extracellular ligands and an arrays of intracellular scaffold proteins [98]. Liprins are intracellular proteins important for transmitting the LAR-RPTPs’ signaling, and serve as scaffolds for assembling large protein complexes to regulate synaptic signaling and assembly.

Accumulating evidence also point to liprins’ roles in cancer, and Pehkonen et al. compiled a list of detailed descriptions in their recent review on this topic [55]. Here, we will focus on the main points for liprins’ roles in cancer. Genetic studies with tumor samples identified alterations in the genes encoding these liprins. Many of these alterations have been associated with several types of cancers, including head and neck squamous cell carcinoma [100,101,102,103,104,105] and breast cancer [106]. These genetic alterations mainly include amplification, mutations, and gene fusions [100,101,102,103,104,105,106,107,108,109,110]. Functional assays have shown that the liprin family plays crucial roles in cell adhesion [111,112,113,114], integrin recycling, and protrusive activity of various cell types [114,115], and these also include tumor cells [53,112]. Indeed, in various cancer cells, liprins can influence cell migration and invasion [53,112,113]. The main oncogenic processes that liprins regulate are likely the cell–cell or cell–substrate interaction, as well as cytoskeleton and the cell membrane composition.

The effects of liprins on cancer cells are context dependent. For example, Liprin-β2 appears to inhibit cell migration and invasion in ovarian and breast cancers [116,117]. On the other hand, Liprin-β1 promotes tumor cell motility and lamellipodia stabilization in breast cancer cells [116]. Liprin-α1 is mostly involved in the promotion of lamellipodia stabilization and invasion in breast [53,112,118,119,120], HNSCC [54,120], colon [121], and bladder cancers [122]. However, it does appear to inhibit invasion in certain conditions in HNSCC [123]. Thus, it is important to better understand the underlying mechanisms for future therapeutic development targeting these proteins.

All three SAMs of Liprin-α have been shown to be required for the binding with D2 domain of LAR [15,30]. They trimerize intramolecularly in a linear head-to-tail fashion to form a unique PPI region, and are stabilized by these interactions as well as by helical linkers and adjacent structures [14,15]. They are involved in the formation of large LAR-RPTP signaling complexes at the cell membrane, localizing the receptors, and bringing in downstream intracellular signal effector proteins. It has not yet been reported if and how these SAMs impact liprins’ cancer roles.

### 5.5. CASKINs

The CASKINs (CASKIN1 and CASKIN2) are CASK (Calcium/Calmodulin Dependent Serine Protein)-interacting proteins. Their structural architecture includes *N*-terminal ankyrin repeats, an SH3 domain, two SAM domains, and an extended Proline-rich *C*-terminal tail [7,19,64]. Similar to liprins, CASKINs can also mediate LAR-RPTP signaling. Interestingly, CASKINs compete with liprin-α for the binding to LAR-RPTP, and play an important role in LAR-dependent axon guidance [124]. Very few studies reported their potential involvement of CASKINs in cancer. A bioinformatic analysis found that CASKIN1 expression is significantly associated with survival of hepatocellular carcinoma and tumor infiltration of multiple immune cell populations [125]. This study utilized TCGA transcriptomic data to assess the tumor purity and tumor infiltration of various cells by calculating immune/stromal/Estimate scores. Another bioinformatic analysis with the TCGA and GEO datasets suggests that CASKIN2 expression is significantly associated with a lower risk with Pancreatic adenocarcinoma (PAAD) [126]. These results suggest that CASKINs have anticancer activities.

There are no reports on functional studies for the roles of CASKIN1/2 in cancer yet, and thus no reports about how the SAM domains affect their roles in cancer. In terms of arrangements of these multi-SAMs, please see Section 3.2.2, where we discussed how these SAMs increase the complexity of SAM-mediated oligomers, through combining various inter- and intra-molecular interactions.

### 5.6. SARM1

SARM1 is an NAD+ hydrolase with scaffolding capacity, and its best-known functions involve axon degeneration and innate immunity, especially the TLR pathways. Very little has been reported regarding its role in cancer. One study with HeLa cells, an HPV-positive cervical cancer cell line, found that knocking out SARM1 decreased cell survival and increased the cells’ sensitivity to cisplatin treatment [127].

The SAMs in SARM1 are important for its functions in axon degeneration [49]. Axons are nerve fibers that extend from the bodies on nerve cells and transmit signals in response to relevant stimuli. Axon degeneration causes loss of communication between neurons and is a common pathological characteristic of many neurodegenerative diseases, such as Alzheimer’s disease and multiple sclerosis. SARM1’s axon degeneration functions depend on its NAD hydrolase activity [128]. SAM domains mediate multimerization that is essential for SARM’s function, and mutations of the SAM domains abolish the ability of SARM to promote axon degradation [49]. For more information on SARM1’s multimerization, please see Section 3.2.2.

Carty and Bowie have proposed compelling models for the role of SARM1 in regulating innate immunity. These models suggest that the activation of SARM1’s diverse functions may arise from shifting of its inactive monomer form to various active forms with distinct functionalities, such as monomer, dimer, or oligomer. The SAM domains may be important for these activations [129], similarly to its function in axon degeneration.

## 6. Conclusions

MSCPs are a group of understudied proteins, especially their roles in cancer. Here, we described how the disordered features of their SAMs and the addition of one or two SAM domains increase the complexity of the interactions and oligomerization of these MSCPs. These results emphasize the importance of studying these SAM domains’ structural dynamics and interaction arrangements. Many structural studies have ignored some phenotypes associated with a disorder feature, such as poor expression of domains/regions in bacteria. We recommend that one should consider exploring these phenotypes further and we hope that our suggested approaches and methods in Section 4 may provide some useful information.

We also identified commonalities among these MSCPs, which include their effects on cancer’s progression and metastasis, and their involvement in various receptor-mediated signaling. Future studies to further understand these functions and regulations will likely lead to a better understanding of the roles of SAM domains and these MCSPs in cancer in general and may lead to new approaches to develop anticancer drugs.

## Figures and Tables

**Table 1:** List of SAM domains functions with select protein examples.

SAM Function	Protein	Reference
Protein–Protein Interaction	SASH3	[22]
	TNKS2	[6]
	Liprins	[15]
Protein–Lipid Interaction	P73	[23]
	Ste11	[11]
Protein–RNA Interaction	Smaug	[12]
